# Breast cancer diagnosis by analysis of serum *N*-glycans using MALDI-TOF mass spectroscopy

**DOI:** 10.1371/journal.pone.0231004

**Published:** 2020-04-09

**Authors:** Sae Byul Lee, Shambhunath Bose, Sei Hyun Ahn, Byung Ho Son, Beom Seok Ko, Hee Jeong Kim, Il Yong Chung, Jisun Kim, Woochang Lee, Myung-Su Ko, Kyungsoo Lee, Suhwan Chang, Hyoung Soon Park, Jong Won Lee, Dong-Chan Kim

**Affiliations:** 1 Division of Breast Surgery, Department of Surgery, Asan Medical Center, University of Ulsan College of Medicine, Seoul, Republic of Korea; 2 R&D Center, NOSQUEST Inc., Seongnam, Gyeonggi, Republic of Korea; 3 Department of Laboratory Medicine, Asan Medical Center, University of Ulsan College of Medicine, Seoul, Republic of Korea; 4 Health Screening and Promotion Center, Asan Medical Center, Seoul, Republic of Korea; 5 Department of Biomedical Sciences, Asan Medical Center, University of Ulsan College of Medicine, Seoul, Republic of Korea; 6 R&D Center, NOSVET Inc., Yongin, Gyeonggi, Republic of Korea; Weill Cornell Medical College in Qatar, QATAR

## Abstract

Blood and serum *N*-glycans can be used as markers for cancer diagnosis, as alterations in protein glycosylation are associated with cancer pathogenesis and progression. We aimed to develop a platform for breast cancer (BrC) diagnosis based on serum *N*-glycan profiles using MALDI-TOF mass spectroscopy. Serum *N*-glycans from BrC patients and healthy volunteers were evaluated using NosQuest’s software “NosIDsys.” BrC-associated “NosID” *N*-glycan biomarkers were selected based on abundance and NosIDsys analysis, and their diagnostic potential was determined using NosIDsys and receiver operating characteristic curves. Results showed an efficient pattern recognition of invasive ductal carcinoma patients, with very high diagnostic performance [area under the curve (AUC): 0.93 and 95% confidence interval (CI): 0.917–0.947]. We achieved effective stage-specific differentiation of BrC patients from healthy controls with 82.3% specificity, 84.1% sensitivity, and 82.8% accuracy for stage 1 BrC and recognized hormone receptor-2 and lymph node invasion subtypes based on *N*-glycan profiles. Our novel technique supplements conventional diagnostic strategies for BrC detection and can be developed as an independent platform for BrC screening.

## Introduction

Early detection of breast cancer (BrC) is associated with more treatment options, better surgical conditions, increased survival, and improved quality of life. While various criteria exist for classification of BrC, it is most commonly classified into non-invasive BrC (stage 0) or invasive ductal carcinoma (IDC; stages 1–4). The presence of hormone receptors (HR) in invasive BrC is a prognostic factor and the most powerful prognostic indicator of hormone suppression. HR is widely used to guide treatment of BrC. If the cancer is estrogen receptor (ER)- or progesterone receptor (PR)-positive, hormonal therapy is the preferred treatment, with or without chemotherapy. According to the HR phenotype, patients are classified as ER+/PR+, ER+/PR-, ER-/PR+, and ER-/PR-. Moreover, based on the expression of the HR and human epidermal growth factor receptor-2 (HER2), BrC can be classified into four major subtypes: luminal 1 (HR+/HER2-), luminal 2 (HR+/HER2+), non-luminal HER2+ (HR-/HER2+), and triple-negative phenotype (HR-/HER2-) [1]. In addition, judging by the penetration of cancer cells into lymph nodes via metastasis, BrC can be classified into two subtypes: without [N (-)] and with [N (+)] lymph node invasion.

The most commonly used diagnostic techniques for BrC include mammography, magnetic resonance imaging, ultrasonography, computerized tomography, positron emission tomography, and biopsy [2]. However, these strategies are expensive, time-consuming, and unsuitable for screening large numbers of patients simultaneously [3,4]. Detecting BrC-specific biomarkers in bodily fluids would be an ideal approach for BrC diagnosis and screening. Alterations in protein glycosylation patterns are potential biomarkers for cancer pathogenesis, metastatic potential, and prognosis [5,6]. Growing evidence indicates differences in glycosylation patterns between tumor cells and healthy cells [5]. More specifically, cancer-related changes in glycosylation are associated with altered expression of glycosyltransferase and chaperone genes, as well as mislocalization of glycosyltransferases [7]. Since some glycoproteins are secreted or shed from tumors, tumor-associated glycan profiles, as well as alterations in protein glycosylation reflecting the host response, can also be detected in serum. In fact, changes in protein glycosylation in serum have been found in various cancer types, including BrC [5,6,8], indicating that serum glycan profiles could be employed as potential biomarkers for BrC.

In this study, we analyzed serum *N*-glycomic patterns based on N stage, which is directly related to biomolecular signatures within bodily fluid. Because we acquired *N*-glycans from patient serum samples, the N stage was primarily considered rather than the overall cancer stage [9].

## Materials and methods

### Subjects and blood collection

The study was approved by the Asan Medical Center (AMC) review board (IRB approval number: 2018–1234). Informed consent was waived. Blood samples were collected from cancer patients and healthy volunteers at AMC (Seoul, South Korea, IRB approval number: 2018–1234). Patient demographics, including, age, disease stage, and tumor cell types, were collected from AMC (Table 1). The collected blood samples were incubated at room temperature and then centrifuged at 1,000 ×*g* for 10 min at room temperature. The supernatant was transferred into 1.5 mL micro-centrifuge tubes (Eppendorf, Hamburg, Germany) and stored at –80 °C.

**Table 1:** Clinical characteristics of patients with BrC.

Classification	Variable	Value	Number of patients (%)
Stage	Stage	I II III IV	113 (44.2) 102 (39.8) 33 (12.9) 8 (3.1)
N stage	Lymph node invasion	Negative Positive	158 (61.7) 98 (38.3)
HR/HER2	Estrogen receptor (ER)	Positive Negative	187 (73.0) 69 (27.0)
	Progesterone receptor (PR)	Positive Negative	154 (60.2) 102 (39.8)
	Tissue HER2 (IHC)	Positive^a^ Negative Unknown	72 (28.3) 182 (71.7) 2

HER2, human epidermal growth factor receptor-2; IHC, immunohistochemistry.

^a^IHC 3+, or IHC 2+ with amplified fluorescence *in situ* hybridization.

### Isolation of *N*-linked glycans

In a microtube, 30 μL serum was mixed with an equal volume of 200 mM NH_4_HCO_3_ containing 1 mM dithiothreitol. Serum proteins were moderately denatured by shaking on a heat block at 65 °C for 5 min at 1,500 rpm. *N*-linked glycans were released from the denatured proteins enzymatically by adding 400 units of peptide-*N*-glycosidase F (PNGase F; New England Biolabs, Ipswich, MA, USA). The PNGase F reaction was carried out in a shaking heat block at 45 °C for 20 min at 1,500 rpm. Then, 540 μL HPLC-grade water and 100 μL 1% TFA were added sequentially. Tubes were centrifuged briefly at 3,000 rpm to spin down the liquid adhering to the inner wall.

### Glycan purification

Glycans released by PNGase F were purified using HyperSep Hypercarb solid-phase extraction (SPE) cartridges fitted on a 96-well plate (Thermo Fisher Scientific, Waltham, MA, USA). The cartridges were packed with 30 μm spherical 100% porous graphitic carbon (PGC) particles and were washed with 1 mL distilled water and 1 mL 80% acetonitrile (ACN)/water (v/v). Prepared *N*-glycan solutions were loaded onto the cartridges and washed three times with 1 mL distilled water. Glycans were eluted in 20% ACN/water (v/v) and fast-dried using a Genevac EZ-2 plus centrifugal vacuum evaporator (Genevac, Valley Cottage, NY, USA). Dried glycans were reconstituted in 15 μL HPLC-grade water for MALDI-TOF mass spectroscopy (MALDI-TOF MS).

### MALDI-TOF MS

Fresh matrix solution was prepared by mixing 2,5-dihydroxybenzoic acid (DHB) (20 mg/mL in ACN) with 40 mM sodium chloride in water at a ratio of 75:25 (v/v). The glycan solution was mixed with the matrix at a ratio of 1:2 (v/v), and 2 μL of the resultant mixture was spotted on an STA μ Focus MALDI target plate (24×16 c 2,000 μm; ASTA, Suwon-si, South Korea). Spotting generated four independent mass spectra per sample. The loaded sample on the MALDI plate was fast-dried in vacuum (6–8 × 10^2^ torr) to facilitate uniform matrix-sample co-crystallization. Mass spectra were acquired using a 4800 Plus MALDI-TOF/TOF MS (AB SCIEX, Framingham, MA, USA) operated in the positive-ion reflection mode, and the *m/z* from 800 to 3,000 was monitored. Glycan mass peaks above 10 S/N (signal-to-noise) were considered valid.

### Data processing and normalization

Mass spectra data were pre-processed, and ion peak information was extracted using 4000 Series Explorer^™^ (Applied Biosystems, Foster City, CA, USA) and in-house software (NosQuest, Seongnam-si, South Korea) that transfers complete peak information (centroid mass values, S/N, heights) from a defined spectrum into a tabulated data format such as Microsoft Excel 2013 (Redmond, WA, USA). Absolute peak intensity (API_*i*_) of each *N*-glycan was normalized to achieve its relative intensity using the following formula:

$${\text{NAPI}}_{\text{i}}=\frac{\text{API}}{\sum_{\text{j}=1}^{\text{n}}\;{\text{API}}_{\text{j}}}$$

where NAPI_*i*_ denotes the normalized absolute peak intensity of a defined *N*-glycan in an acquired mass spectrum, and *n* represents the number of total peaks in the mass spectrum (total ion chromatogram). Each API was divided by the sum of total APIs in the spectrum (total ion current; TIC). For convenience, NAPI values were multiplied by 1,000.

### Visualization and statistical analyses

The normalized intensity data for the *N*-glycans corresponding to *N*-glycan species obtained from the NosQuest proprietary biomarker panel were gathered and converted to TSV files using Microsoft Excel. These were then analyzed using Perseus^™^ (Max Planck Institute of Biochemistry, Berlin, Germany). A multiple-sample test was performed using analysis of variance (ANOVA) with a *P* value truncation method, with the threshold *P* value set to 0.05. In *Z* score normalization, each data point was subtracted from the mean value of the aggregate data and then divided by the standard deviation of the total data [10]. Perseus^™^ 1.5.2.6 was used for hierarchical clustering, principal component analysis (PCA), and plotting [11]. Acquired peaks were initially filtered using an *m/z* ratio range of 900 to 3,000. Next, *m/z* features were filtered based on a reference glycan list of 239 known human glycans [12]. For each *m/z*, four replicate data points were averaged only if more than two out of the four values were present. Otherwise, they were assigned as Not-a-Number or 0. The *m/z* features were then filtered a second time using a cut-off frequency of more than 90% of existing values across the samples. The *m/z* features were then analyzed by multiple-sample ANOVA with a *P* value cut-off of 0.05.

**Fig 1:**
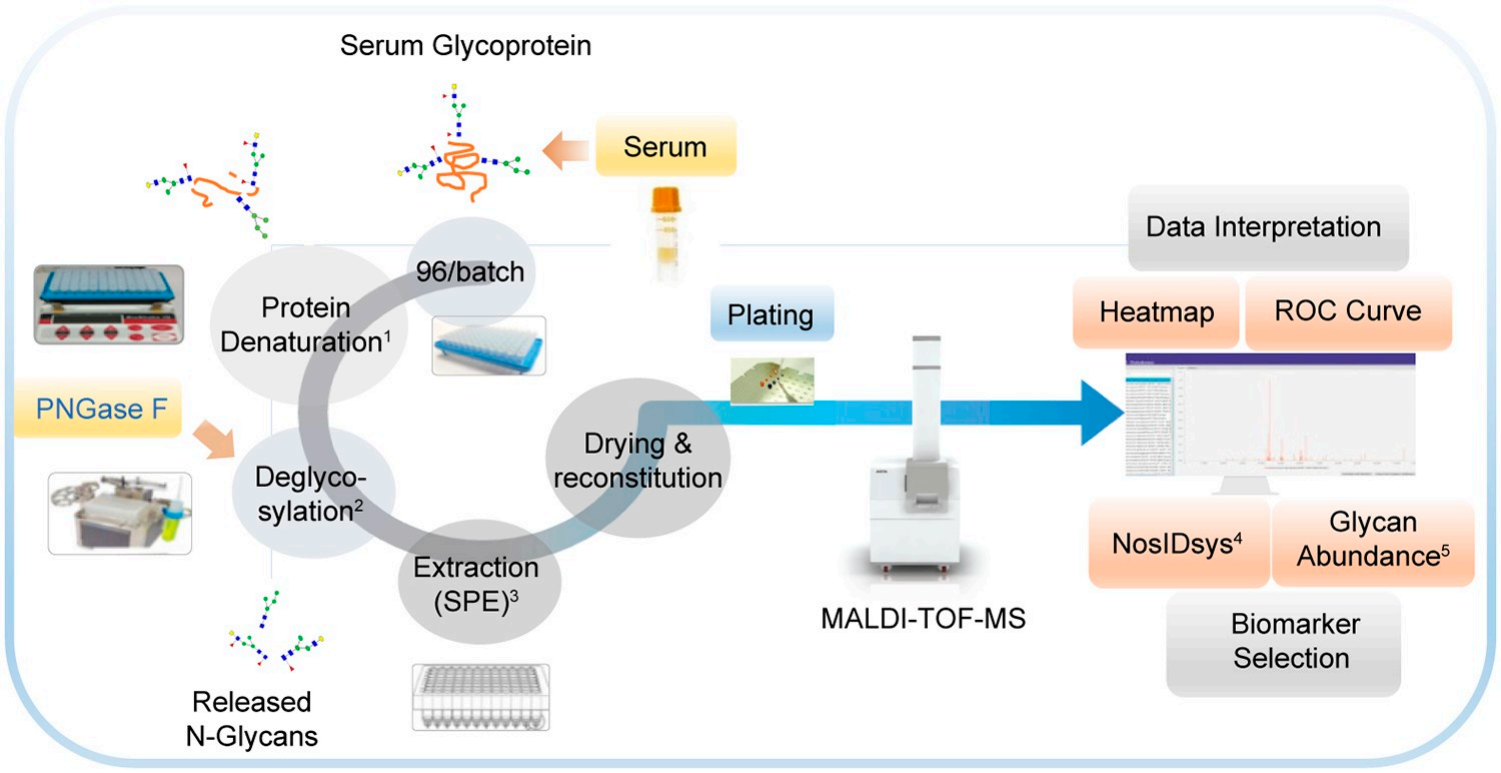
Schematic representation of procedures for the extraction, purification, and MALDI-TOF MS of serum *N*-glycans and analysis of data. ^1^Denaturation, ^2^deglycosylation, ^3^solid-phase extraction (SPE), ^4^NosIDsys, and ^5^glycan abundance.

Normalized intensity data of *N*-glycans corresponding to *N*-glycan species from the NosQuest proprietary biomarker panel were extracted and converted to CSV files in which all cluster markers that were selected in the heat map analysis were filtered, saved, and used to construct the receiver operating characteristic (ROC) curve. A schematic depiction of the procedure for serum *N*-glycan preparation, analysis, and data processing is shown in Fig 1.

## Results

### Normal versus IDC MALDI-TOF signature

**Fig 2:**
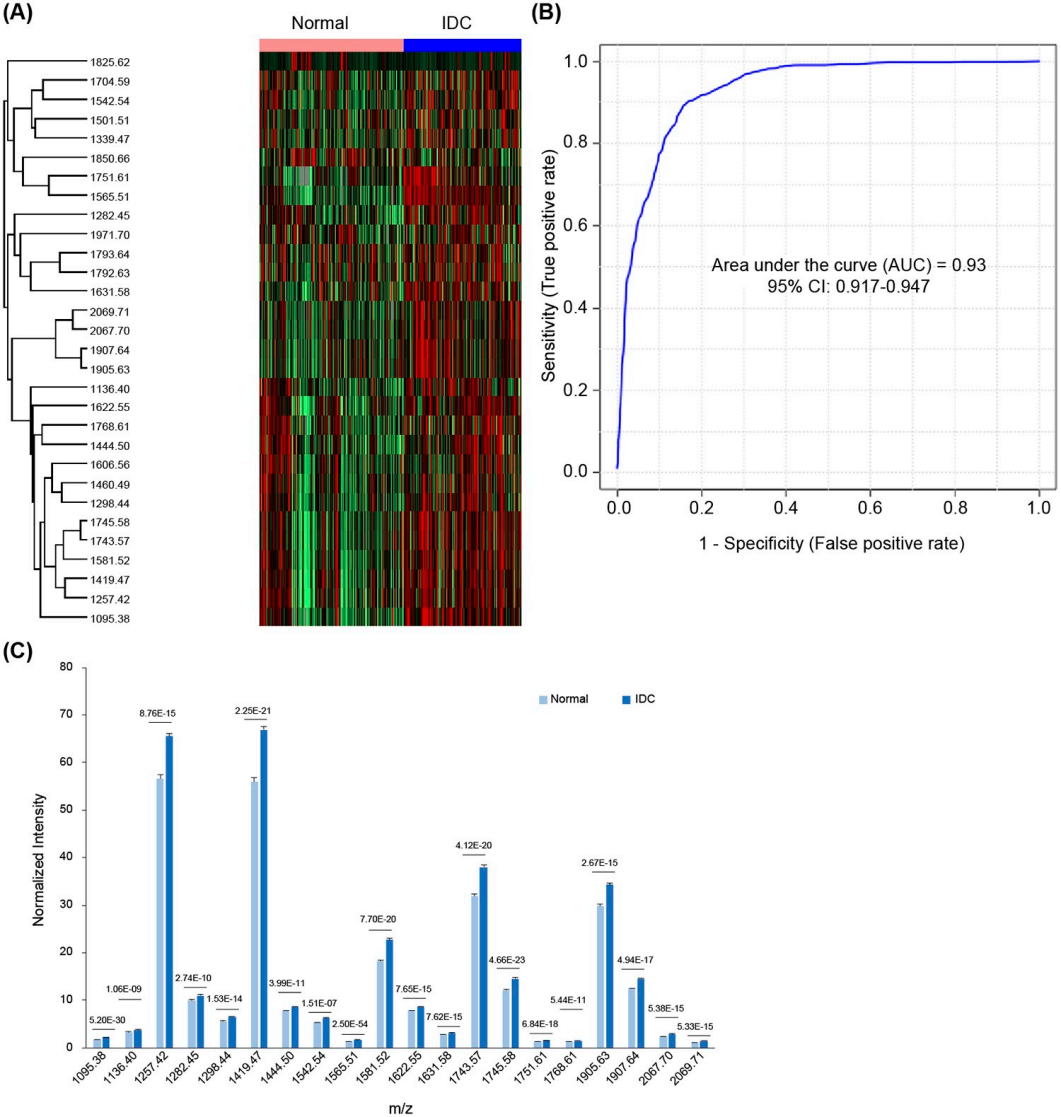
Comparison of the healthy and IDC subjects via heat map, ROC, and normalized intensity. (A) Heat map showing expression profiles of selected serum *N*-glycans between healthy and IDC participants. Red, higher relative expression; green, lower relative expression. (B) ROC curve showing the diagnostic performance in distinguishing IDC patients from healthy subjects (C) Mean normalized intensity versus *m/z* of NosID *N*-glycan biomarkers between normal and IDC serum samples. *P* values for differences between the two groups are depicted above the corresponding bars. Error bars represent standard deviations.

**Fig 3:**
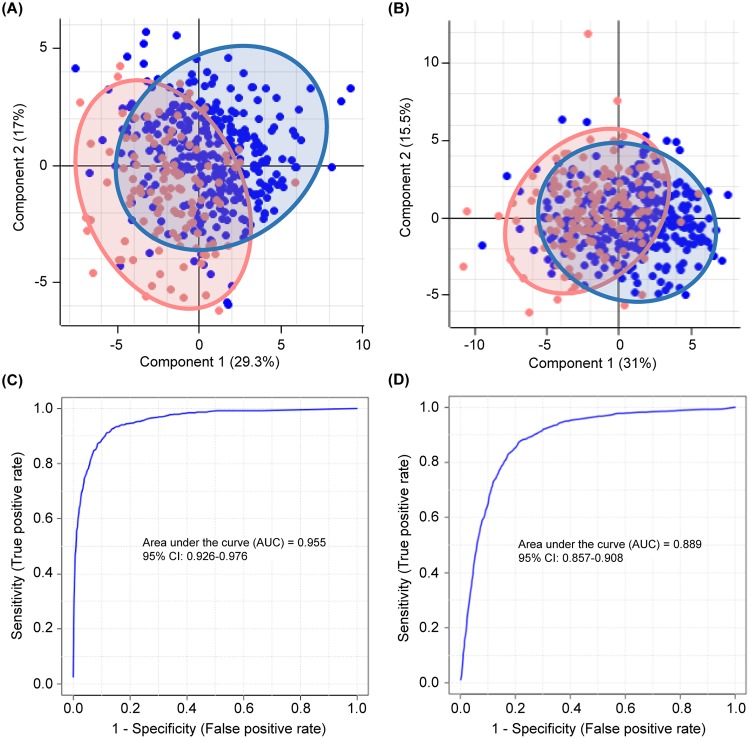
Comparison of the normal and BrC stages via PCA and ROC. PCA plots (A, B) and ROC curves (C, D) for healthy controls vs. stage 1 (A, C) and stage 2–4 (B, D) BrC samples. Blue and pink spots indicate individual samples within the healthy control and terminal BrC groups, respectively.

Serum *N*-glycan profiles were studied using MALDI-TOF MS in healthy volunteers (n = 311) and subjects with IDC (n = 256), including BrC stage 1 (BrC1; n = 113), stage 2 (BrC2, n = 102), stage 3 (BrC3, n = 33), and stage 4 (BrC4, n = 8). The average ages of these groups were 50.88, 51.93, 48.00, 50.24, and 54.75, respectively, and all samples were acquired from females. Following mass analysis, *N*-glycan peak intensities were normalized with the TIC to obtain relative intensities. The heat map showed significant differential expression of 30 *N*-glycan species from NosQuest’s proprietary *N*-glycan biomarker panel in the sera of IDC patients compared to that in healthy individuals (Fig 2A). We observed very high diagnostic efficacy of these filtered biomarkers in differentiating IDC patients from healthy subjects as reflected by an area under the curve (AUC) of 0.93 [95% confidence interval (CI): 0.917–0.947; Fig 2B, S1 Fig]. We then extracted 20 *N*-glycans from NosQuest’s *N*-glycan panel. These filtered glycan biomarkers exhibited higher accuracy in NosIDsys screening and a ≥10% difference in abundance based on normalized intensity between healthy and IDC subjects. These “NosID biomarkers” showed significantly higher expression in IDC patients than in healthy controls (Fig 2C). Levels of significance of these differences and annotations of the associated biomarkers are depicted in Table 2. Specific *N*-glycan markers for each BrC stage and subtype were assigned (Table 2). *N*-glycans 1136.401 and 1339.467 (M+Na) were markers of stage 1, while 1606.558, 1768.610, 1444.499, 1460.495, and 1662.550 (M+Na) were dominant only in stages 2–4. By NosIDsys analysis comparing normal and stage 1 samples, specificity, sensitivity, and accuracy values of 82.3%, 84.1%, and 82.8% were obtained, while normal versus stage 2–4 samples showed values of 73.5%, 75.2%, and 74.0%, respectively. By PCA, we observed separate clusters of normal samples and all BrC stages combined, showing that the *N*-glycomic patterns differentiated the two groups. PCA was performed separately for normal versus stage 1 (Fig 3A) and normal versus stages 2–4 (Fig 3B). In both cases, sample groups formed unique clusters, suggesting that normal and BrC samples are distinguishable regardless of stage. ROC curves were plotted and corresponding AUCs calculated. As shown in Fig 3C and 3D, analysis of stage 1 showed better performance than stages 2–4, with AUCs of 0.955 and 0.889, respectively.

**Table 2:** *N*-glycans with significantly different MALDI-TOF intensities in BrC patients and healthy controls.

**Mass** **(M+Na)**	**1095.379**	**1136.401**	**1282.422**	**1298.441**	**1339.467**	**1542.538**	**1565.514**	**1631.580**	**1745.580**	**1751.608**	**1606.558**	**1768.610**	**1793.636**
**BrC** **Stage**	**Stage 1** **Stage 2–4**	**Stage 1**	**Stage 1** **Stage 2–4**	**Stage 1** **Stage 2–4**	**Stage 1**	**Stage 1** **Stage 2–4**	**Stage 1** **Stage 2–4**	**Stage 1** **Stage 2–4**	**Stage 1** **Stage 2–4**	**Stage 1** **Stage 2–4**	**Stage 2–4**	**Stage 2–4**	
**BrC** **Subtype**	**HR+/HER2+** **HR+/HER2-** **HR-/HER2+** **HR-/HER2-** **N(+), N(-)**	**HR+/HER2+** **HR+/HER2-** **HR-/HER2-** **N(-)**	**HR+/HER2-** **HR-/HER2-** **N(-)**	**HR+/HER2+** **HR+/HER2-** **HR-/HER2+** **HR-/HER2-** **N(+), N(-)**	**HR+/HER2+** **HR-/HER2+** **HR-/HER2-** **N(+), N(-)**	**HR+/HER2+** **HR-/HER2+** **HR-/HER2+** **HR-/HER2-** **N(+), N(-)**	**HR+/HER2+** **HR+/HER2-** **HR-/HER2+** **HR-/HER2-** **N(+), N(-)**	**HR+/HER2-** **HR-/HER2-** **N(+), N(-)**	**HR+/HER2+** **HR+/HER2-** **HR-/HER2+** **N(+), N(-)**	**HR+/HER2+** **HR+/HER2-** **HR-/HER2+** **HR-/HER2-** **N(+), N(-)**	**HR+/HER2+** **HR-/HER2+** **HR-/HER2+** **N(+)**	**HR+/HER2-** **HR-/HER2+** **N(+), N(-)**	**HR+/HER2-**
**Mass** **(M+Na)**	**1907.639**	**2067.698**	**2069.711**	**1257.422**	**1419.470**	**1581.520**	**1743.573**	**1905.631**	**1444.499**	**1460.495**	**1622.550**	**1704.585**	
**BrC** **Stage**	**Stage 1** **Stage 2–4**	**Stage 1** **Stage 2–4**	**Stage 1** **Stage 2–4**	**Stage 1** **Stage 2–4**	**Stage 1** **Stage 2–4**	**Stage 1** **Stage 2–4**	**Stage 1** **Stage 2–4**	**Stage 1** **Stage 2–4**	**Stage 2–4**	**Stage 2–4**	**Stage 2–4**		
**BrC** **Subtype**	**HR+/HER2+** **HR+/HER2-** **HR-/HER2+** **N(+), N(-)**	**HR+/HER2+** **HR+/HER2-** **HR-/HER2+** **HR-/HER2-** **N(+), N(-)**	**HR+/HER2+** **HR+/HER2-** **HR-/HER2+** **HR-/HER2-** **N(+), N(-)**	**HR+/HER2+** **HR+/HER2-** **HR-/HER2+** **N(+), N(-)**	**HR+/HER2+** **HR+/HER2-** **HR-/HER2+** **N(+), N(-)**	**HR+/HER2+** **HR+/HER2-** **HR-/HER2+** **N(+), N(-)**	**HR+/HER2+** **HR+/HER2-** **HR-/HER2+** **N(+), N(-)**	**HR+/HER2+** **HR+/HER2-** **HR-/HER2+** **N(+), N(-)**	**HR+/HER2-** **HR-/HER2-** **N(+)**	**HR+/HER2+** **HR+/HER2-** **HR-/HER2+** **N(+), N(-)**	**HR-/HER2+** **N(+)**	**HR-/HER2+** **N(+)**	

### Normal versus BrC subtype signatures

For analyzing the *N*-glycomic signatures across multiple subtypes, we took HR, HER2, and N stage into account. Similarly, HR and HER2 factors were chosen since they represent classification methods directly based on biomolecular abundance. Twenty-five NosID glycan biomarkers were identified for differentiating healthy volunteers from the four HR/HER2 subtypes of BrC (Table 2). The abundances of all respective biomarkers for each subtype were significantly higher than those in the healthy group (S3–S6 Figs). In particular, 1793.636 (M+Na) differentiated HR+/HER2- from healthy controls. Twenty-four NosID glycan biomarkers distinguished healthy volunteers from BrC without [N (-)] and with [N (+)] lymph node invasion (Table 2). The expression levels of all biomarkers corresponding to either N (-) or N (+) were significantly higher than those in the healthy group (S7 and S8 Figs). Overall, 25 *N*-glycans were selected as biomarker candidates for BrC stages and subtypes. Those with *m/z* of 1622.550, 1704.585, and 1793.636 showed significant differences in MALDI-TOF intensity between BrC patients and healthy controls, as indicated in Table 2.

## Discussion

*N*-glycans play critical roles in the initiation and progression of cancer [13]. Alterations in protein glycosylation in serum have been observed in several cancers, including BrC [5,6,8,14], suggesting that serum glycans could be potential biomarkers for BrC. Based on MALDI-TOF MS of human serum *N*-glycans, we developed multi-biomarker panels for screening BrC patients at different stages of progression, lymph node invasion, and HR/HER2 expression. More specifically, we established a screening system using NosQuest’s proprietary software “NosIDsys.” In this procedure, glycan biomarkers were selected based on expression or abundance in terms of normalized peak intensities (with a cut-off value of a 10% difference in normalized intensities between the healthy and BrC groups) and NosIDsys analysis.

Interestingly, early-stage cancer samples showed a higher AUC than that of stages 2–4. Although it is difficult to provide a clear explanation for this result, it seems that BrC-specific *N*-glycomic signatures are dominant during the relatively early stages, rather than in terminal cancer. Our results provide positive insight into the feasibility of *N*-glycome-based methods for the early diagnosis of BrC.

Our results are also in agreement with previous observations in human serum samples, which revealed that the abundances of the high-mannose glycans Hex6HexNAc2, Hex7HexNAc2, Hex9HexNAc2, and Hex10HexNAc2 were significantly higher in BrC patients than in healthy individuals [15,16]. Accordingly, most mature glycoproteins departing from the Golgi complex carry *N*-glycans, while most present in the EPR are still attached to high-mannose glycans [17]. Altered expression of glycosyltransferase genes is thought to be a predominant contributor to differential changes in cellular glycan structures and is therefore considered a hallmark of neoplastic cell metamorphoses [18]. Thus, it is conceivable that elevated levels of high-mannose glycans alter protein stability, adhesion, and communication, thereby contributing to the genesis and growth of BrC cells.

Among IDC patients, 81% were HR+, while 28% were HER2+. Furthermore, 63% of HER2+ patients were HR+/HER2+. HER2, a member of the Erb family, promotes oncogenic transformation and tumor growth [19]. Nearly 75% of BrCs express ER and/or PR, while approximately 20% of BrCs exhibit overexpression or amplification of HER2. Moreover, about 63% of HER2+ BrCs co-express ER/PR [20].

While the promising ability of early diagnosis is important in terms of practical feasibility in clinical applications, the ability to classify samples into various pre-existing subgroups is also important in terms of treatment. Thus, we attempted to provide evidence of the linkage between *N*-glycomic characteristics and various subgroups. In our study, we found that one complex/hybrid glycan (*m/z* 1444.499) and four hybrid glycan members (*m/z* 1460.495, 1606.558, 1622.550, and 1768.610) were biomarkers for distinguishing BrC2–4 patients from healthy participants. In an earlier report, the accuracy (AUC) of Hex5HexNAc3dHex1 (*m/z* 1606.558) and Hex6HexNAc3 (*m/z* 1622.550) glycans in differentiating epithelial ovarian cancer (EOC) stages 3–4 from healthy controls was higher than their accuracy in segregating EOC stages 1–2 from healthy controls (S2 Fig) [16]. Accordingly, a hybrid glycan (*m/z* 1622.550) and a complex/hybrid glycan (*m/z* 1704.585) were discovered to serve as biomarkers for differentiating HR-/HER2+ from healthy controls, and a complex/hybrid glycan (*m/z* 1793.636) was a specific biomarker for differentiating HR+/HER2- from healthy controls.

## Conclusions

We identified 24 NosID glycan biomarkers for differentiating healthy volunteers from N (-) and N (+) BrC subtypes. Notably, the differential expression of *N*-glycans between N (-) and N (+) subtypes was more pronounced for complex, complex/hybrid, and hybrid glycans compared to that for high-mannose glycans. Increased activity or expression of *N*-acetylglucosaminyltransferase V (MGAT5) and β-1,6 GlcNAc-branched *N*-glycans has been observed in highly metastatic tumors, including BrC [21]. In contrast, a study evaluating specific metastasis-related *N*-glycan alterations in EOC has reported that a decrease in bisecting GlcNAc structure is related to higher metastatic potential [22]. Our results demonstrate that *N*-glycomic analysis of BrC using MALDI-TOF has a higher diagnostic efficiency than that of conventional strategies such as mammography or ultrasonography. Specifically, sensitivity values in a previous study were 66.7% and 33.3% for ultrasonography and mammography, respectively [23]. In this study, sensitivity between normal and stage 1 BrC samples reached 84.1%, suggesting that *N*-glycomics is a promising strategy for fast and sensitive early BrC diagnosis in the clinic. However, this result relies on statistical analysis based on a limited number of BrC and healthy samples. Thus, the representativeness of the enlisted glycan markers should be further evaluated using a larger cohort.

## Supporting information

S1 Fig Mean normalized intensity versus the mass-to-charge ratio of NosID N-glycan biomarkers between healthy controls and stage 1 serum samples. *P* values for the difference in outcome rates of the normalized intensities of glycan peaks between the two groups are depicted above the corresponding bars. Error bars represent standard deviations.
(TIF)

S2 Fig Mean normalized intensity versus the mass-to-charge ratio of NosID N-glycan biomarkers between healthy controls and stage 2–4 serum samples. *P* values for the difference in outcome rates of the normalized intensities of glycan peaks between the two groups are depicted above the corresponding bars. Error bars represent standard deviations.
(TIF)

S3 Fig Mean normalized intensity versus the mass-to-charge ratio of NosID N-glycan biomarkers between healthy controls and HR+/HER2- subtype serum samples. *P* values for the difference in outcome rates of the normalized intensities of glycan peaks between the two groups are depicted above the corresponding bars. Error bars represent standard deviations.
(TIF)

S4 Fig Mean normalized intensity versus the mass-to-charge ratio of NosID N-glycan biomarkers between healthy controls and HR+/HER2+ subtype serum samples. *P* values for the difference in outcome rates of the normalized intensities of glycan peaks between the two groups are depicted above the corresponding bars. Error bars represent standard deviations.
(TIF)

S5 Fig Mean normalized intensity versus the mass-to-charge ratio of NosID N-glycan biomarkers between healthy controls and HR-/HER2+ subtype serum samples. *P* values for the difference in outcome rates of the normalized intensities of glycan peaks between the two groups are depicted above the corresponding bars. Error bars represent standard deviations.
(TIF)

S6 Fig Mean normalized intensity versus the mass-to-charge ratio of NosID N-glycan biomarkers between healthy controls and HR-/HER2- subtype serum samples. *P* values for the difference in outcome rates of the normalized intensities of glycan peaks between the two groups are depicted above the corresponding bars. Error bars represent standard deviations.
(TIF)

S7 Fig Mean normalized intensity versus the mass-to-charge ratio of NosID N-glycan biomarkers between healthy controls and N (-) serum samples. *P* values for the difference in outcome rates of the normalized intensities of glycan peaks between the two groups are depicted above the corresponding bars. Error bars represent standard deviations.
(TIF)

S8 Fig Mean normalized intensity versus the mass-to-charge ratio of NosID N-glycan biomarkers between healthy controls and N (+) serum samples. *P* values for the difference in outcome rates of the normalized intensities of glycan peaks between the two groups are depicted above the corresponding bars. Error bars represent standard deviations.
(TIF)
