# Supplementary figures and images for: Breast cancer diagnosis by analysis of serum N-glycans using MALDI-TOF mass spectroscopy
Source: PLoS One. 2020 Apr 9;15(4):e0231004. doi: 10.1371/journal.pone.0231004 (PMC7144955; doi:10.1371/journal.pone.0231004)

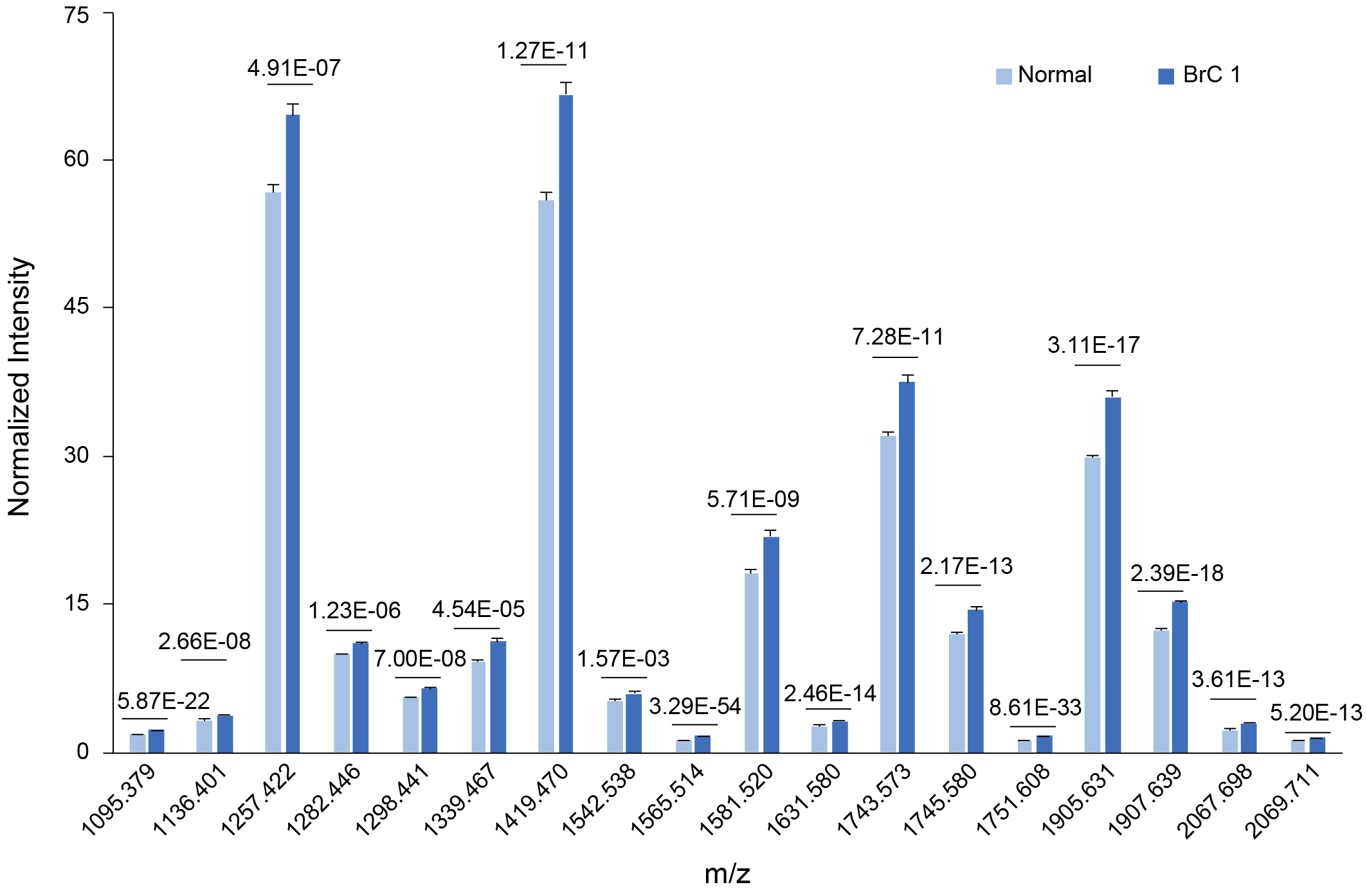

Supplement: S1 Fig — P values for the difference in outcome rates of the normalized intensities of glycan peaks between the two groups are depicted above the corresponding bars. Error bars represent standard deviations. (TIF) [file pone.0231004.s001.tif]

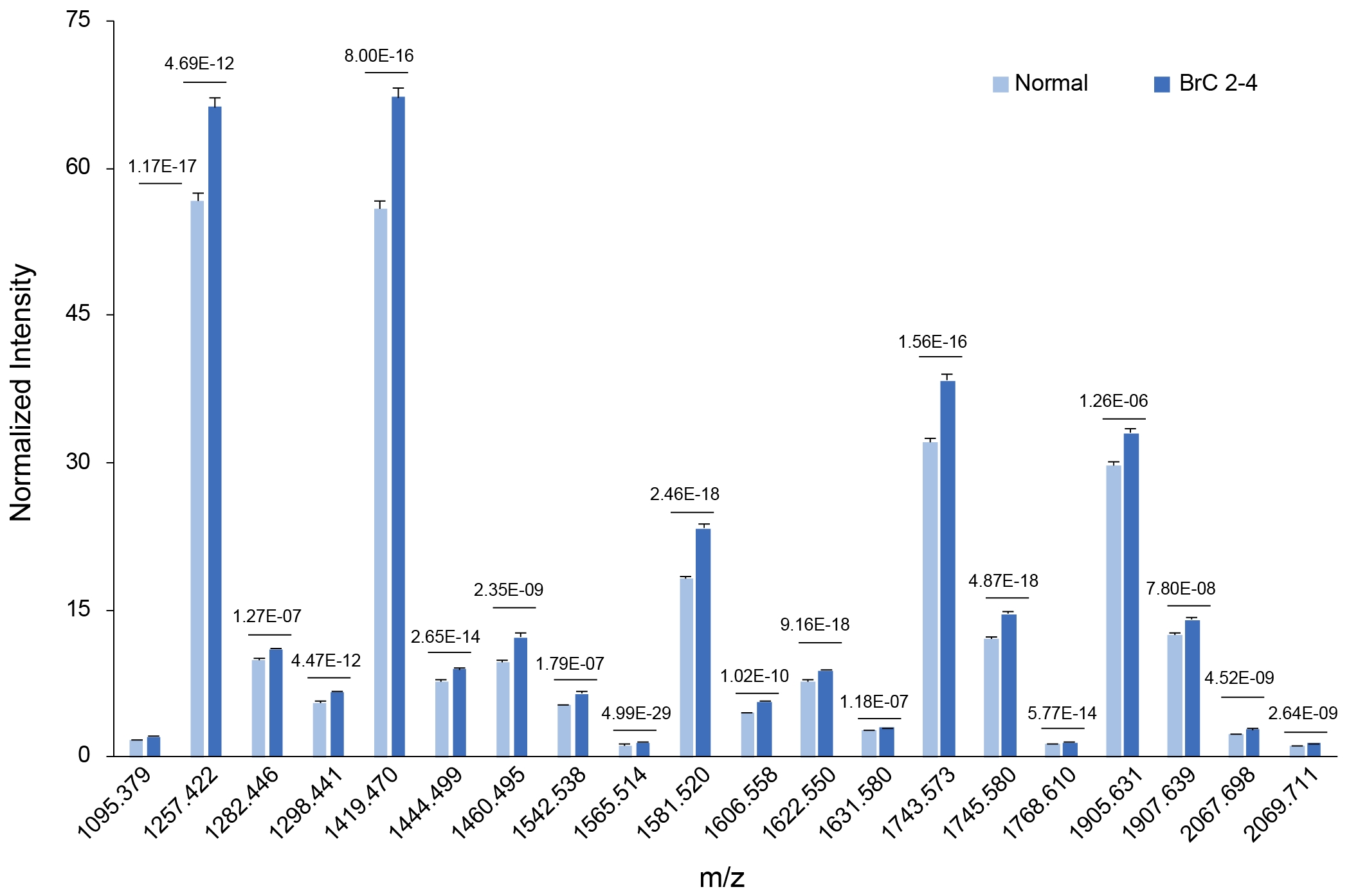

Supplement: S2 Fig — P values for the difference in outcome rates of the normalized intensities of glycan peaks between the two groups are depicted above the corresponding bars. Error bars represent standard deviations. (TIF) [file pone.0231004.s002.tif]

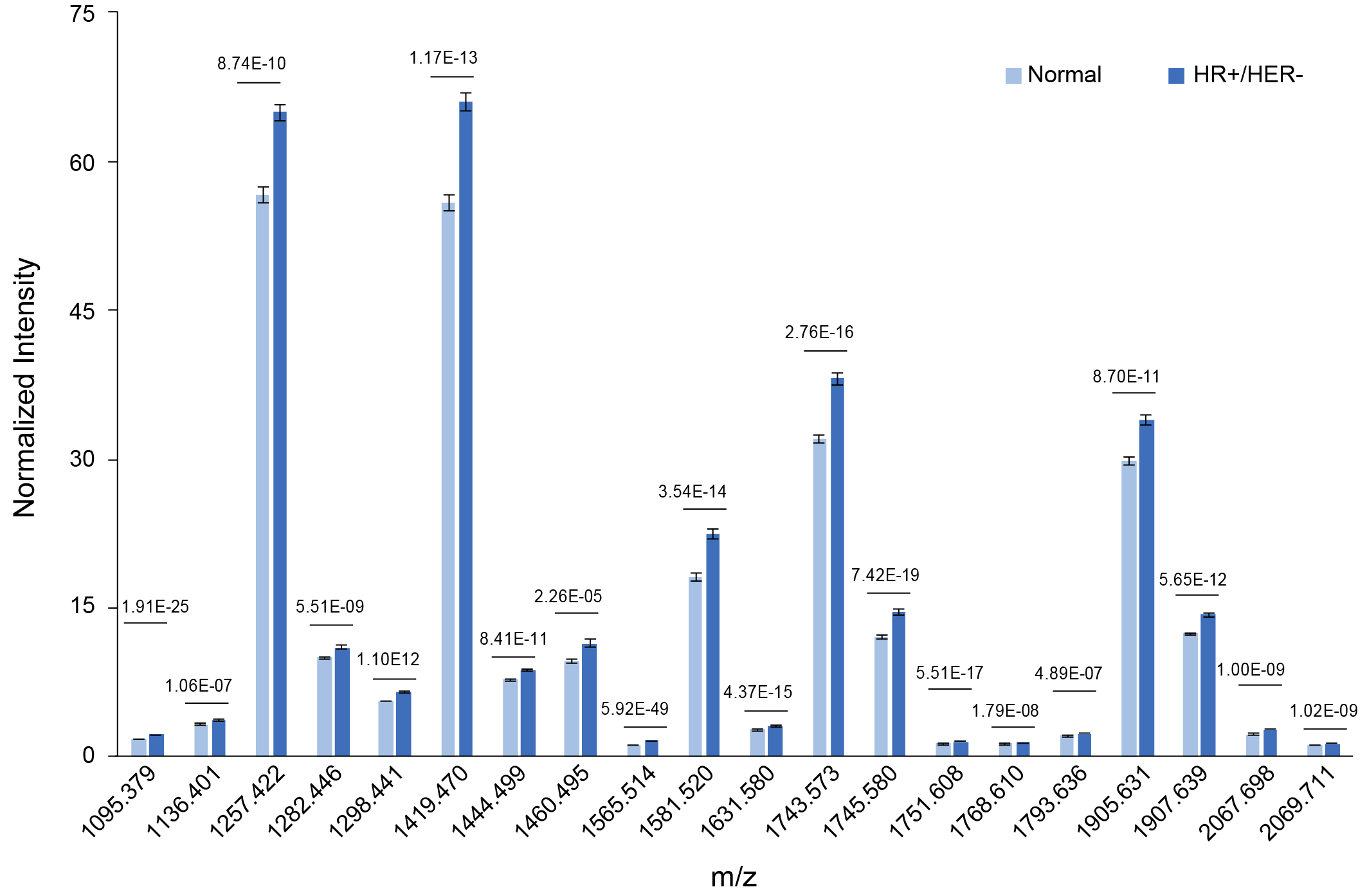

Supplement: S3 Fig — P values for the difference in outcome rates of the normalized intensities of glycan peaks between the two groups are depicted above the corresponding bars. Error bars represent standard deviations. (TIF) [file pone.0231004.s003.tif]

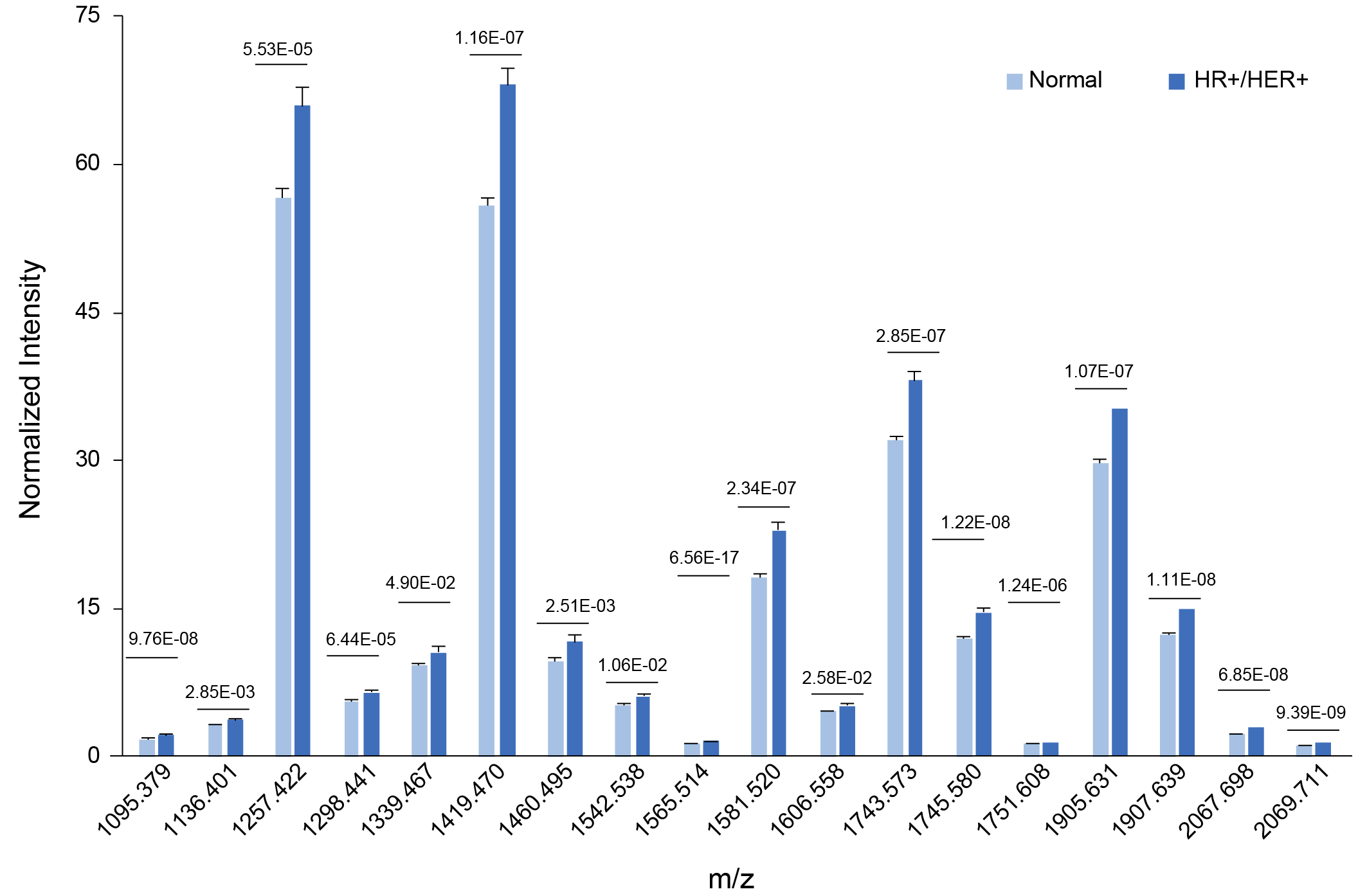

Supplement: S4 Fig — P values for the difference in outcome rates of the normalized intensities of glycan peaks between the two groups are depicted above the corresponding bars. Error bars represent standard deviations. (TIF) [file pone.0231004.s004.tif]

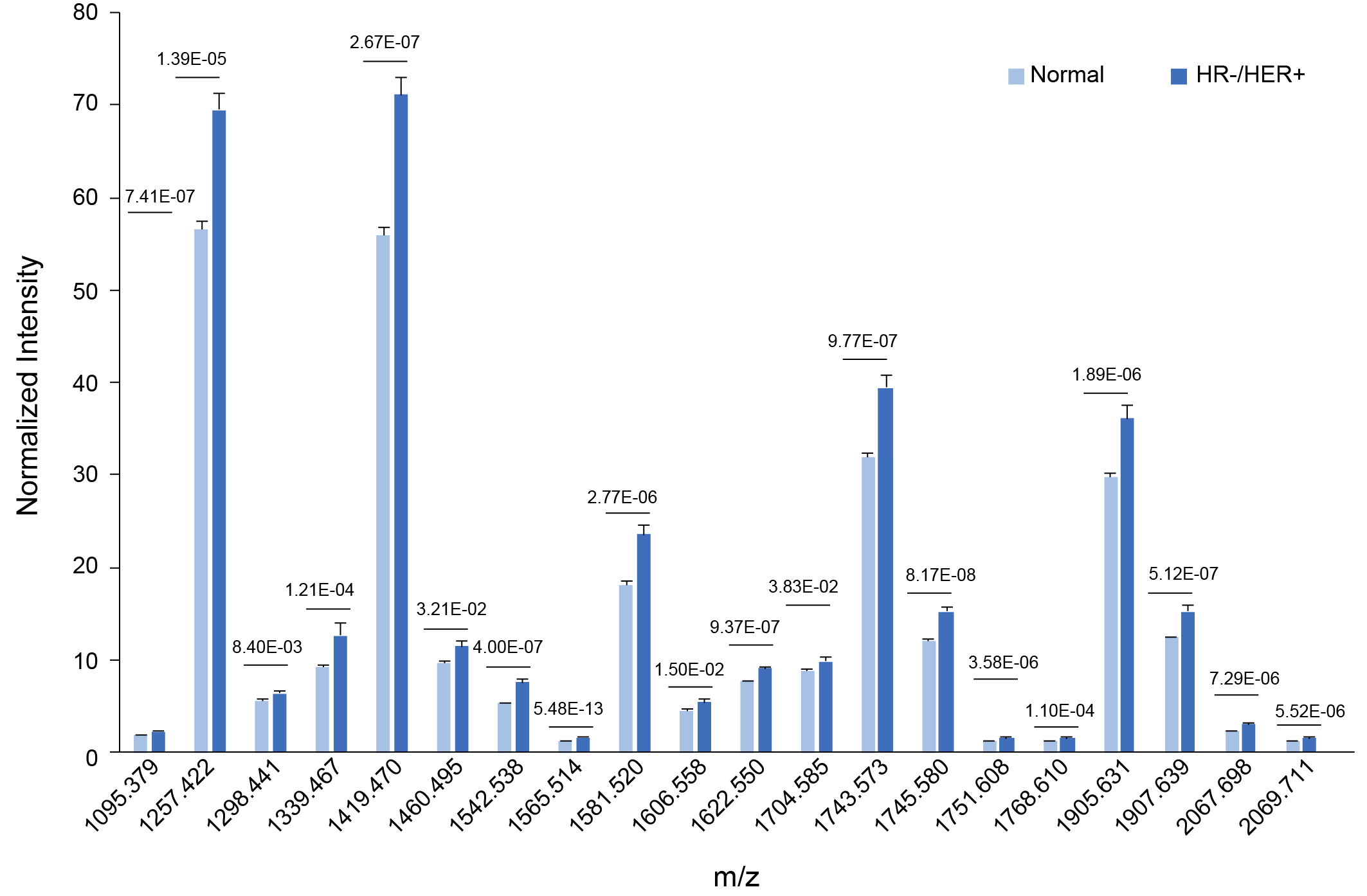

Supplement: S5 Fig — P values for the difference in outcome rates of the normalized intensities of glycan peaks between the two groups are depicted above the corresponding bars. Error bars represent standard deviations. (TIF) [file pone.0231004.s005.tif]

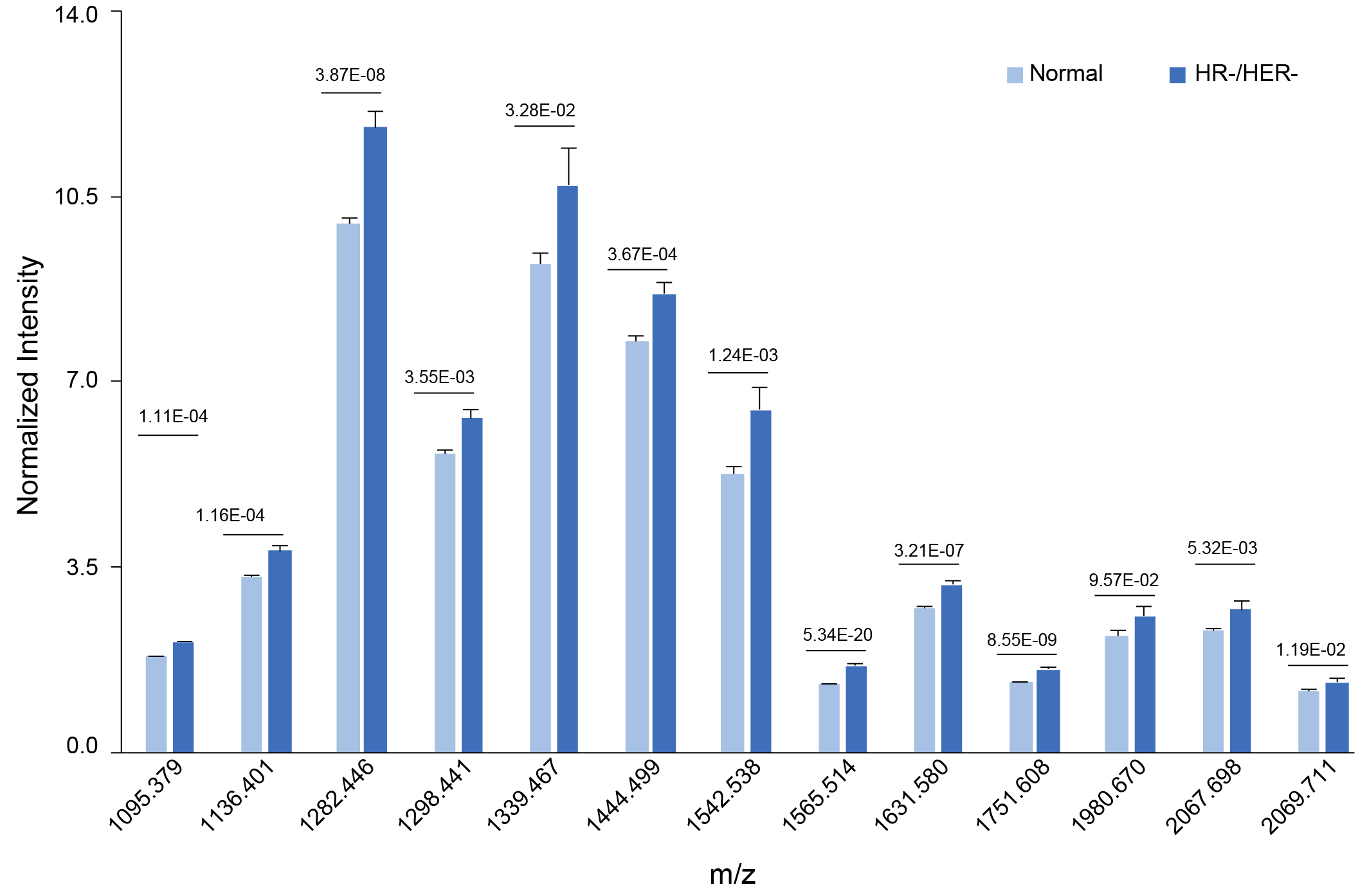

Supplement: S6 Fig — P values for the difference in outcome rates of the normalized intensities of glycan peaks between the two groups are depicted above the corresponding bars. Error bars represent standard deviations. (TIF) [file pone.0231004.s006.tif]

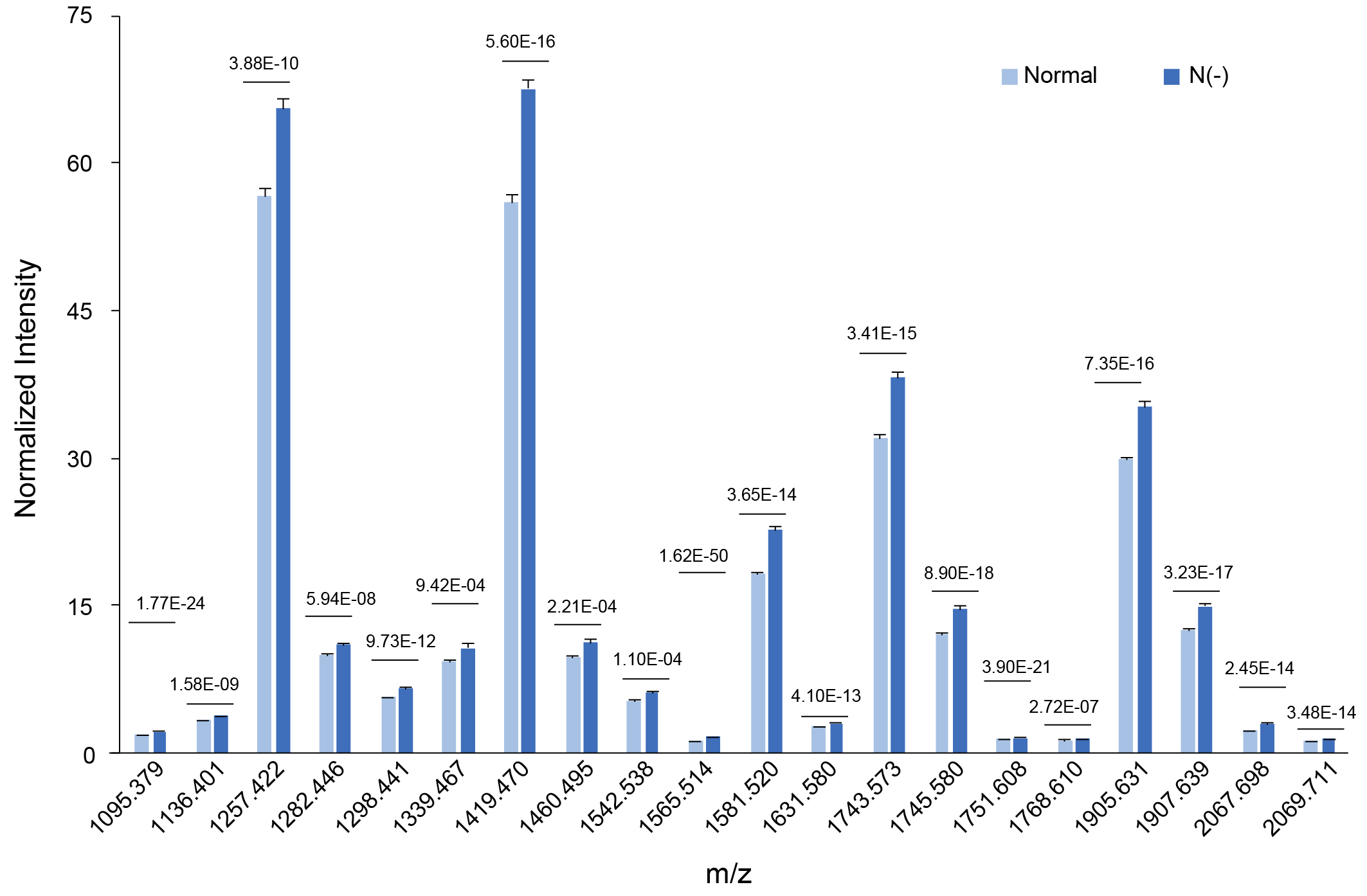

Supplement: S7 Fig — P values for the difference in outcome rates of the normalized intensities of glycan peaks between the two groups are depicted above the corresponding bars. Error bars represent standard deviations. (TIF) [file pone.0231004.s007.tif]

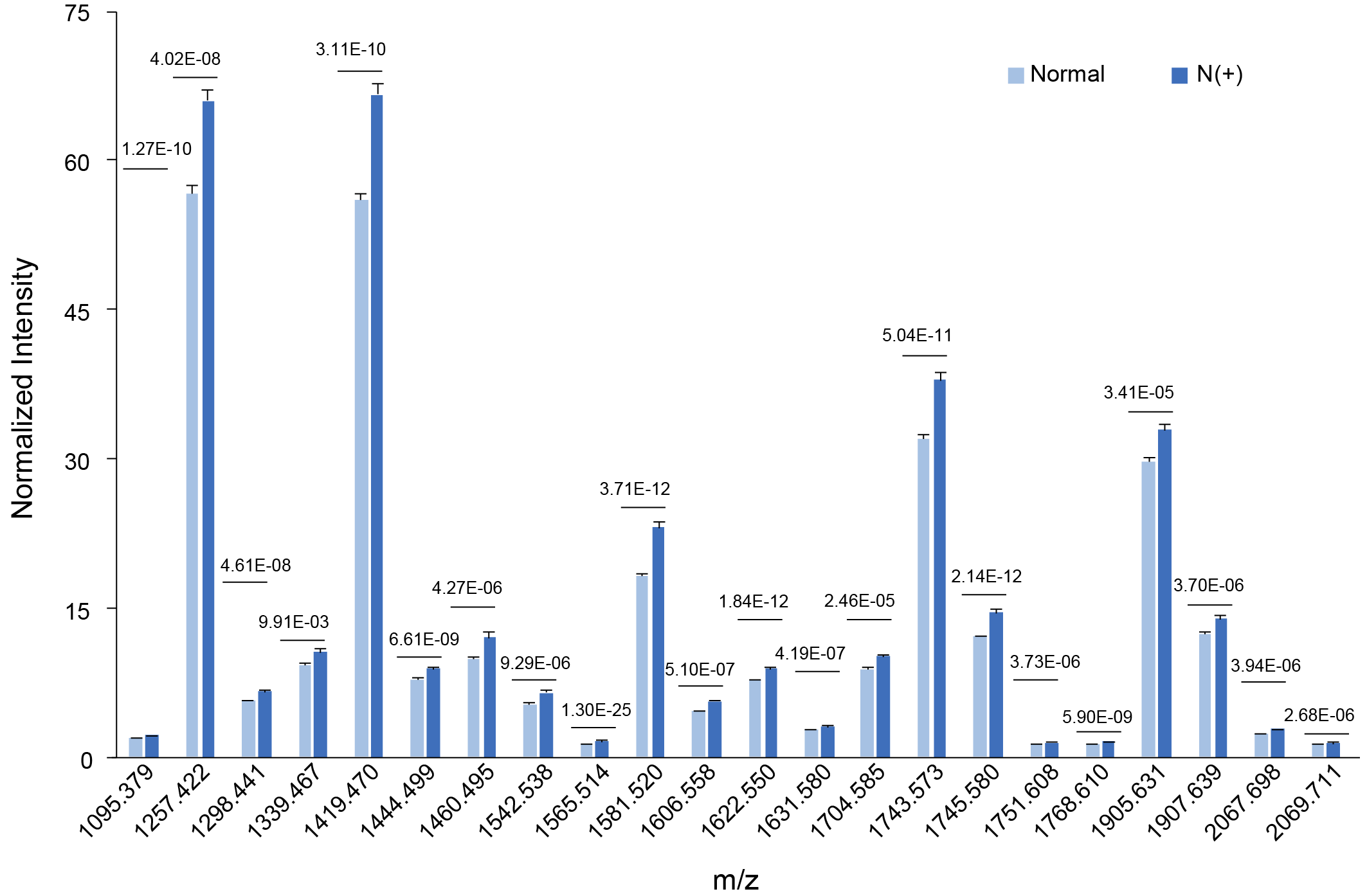

Supplement: S8 Fig — P values for the difference in outcome rates of the normalized intensities of glycan peaks between the two groups are depicted above the corresponding bars. Error bars represent standard deviations. (TIF) [file pone.0231004.s008.tif]
